# Functional pancreatic neuroendocrine tumour causing Cushing’s syndrome: the effect of chemotherapy on clinical symptoms

**DOI:** 10.3332/ecancer.2017.773

**Published:** 2017-10-13

**Authors:** Paulo Henrique do Amor Divino, Katia Regina Marchetti, Madson Q Almeida, Rachel P Riechelmann

**Affiliations:** 1 Instituto do Câncer do Estado de São Paulo, Av Dr Arnaldo, 251 - Cerqueira César, Sao Paulo, 1246-000, Brazil; 2 AC Camargo Cancer Center, Sao Paulo 01509-900, Brazil

**Keywords:** neuroendocrine tumours, Cushing syndrome, pancreatic neoplasms

## Abstract

**Background:**

Neuroendocrine tumours (NETs) are a heterogeneous group of diseases that can originate from any part of the gastrointestinal tract, bronchi, thyroid and pancreas. These tumours may be functioning or not depending on their ability to produce active substances, such as adrenocorticotrophic hormone (ACTH). ACTH-producing pancreatic neuroendocrine tumours are rare, with limited data about effective antitumor therapies.

**Case Report:**

A 58-year-old man with a history of type-2 diabetes mellitus and arterial hypertension was diagnosed with Cushing’s syndrome (CS) secondary to an ACTH ectopic production from a well-differentiated neuroendocrine tumour of the pancreas metastatic to the liver. The patient underwent initial body-caudal pancreatectomy, splenectomy and hepatic nodulectomy with subsequent recurrence. Hepatic embolisation and somatostatin analogues were used to control CS but without success. Bilateral adrenalectomy led to CS control, while capecitabine and oxaliplatin (CAPOX) was effective in controlling tumour growth and ACTH production.

**Discussion:**

ACTH-producing pancreatic neuroendocrine tumours are rare, aggressive and difficult to treat with available therapies. In settings of limited resources, such as in developing countries where targeted therapies are not available, cytotoxic chemotherapy with CAPOX represents a good and inexpensive option to control ACTH-producing pancreatic neuroendocrine tumours. Because of its complexity, the management of this tumour should be performed by multidisciplinary teams.

## Introduction

Neuroendocrine tumours (NETs) are a heterogeneous group of diseases that can originate in any part of the gastrointestinal tract, bronchi, thyroid and pancreas [1]. They are rare diseases and probably underdiagnosed, since in many cases the course of the disease is indolent. Functioning pancreatic NETs (PNETs) are rare, representing approximately 1% of pancreatic neoplasias [2, 3]. Among these tumours, those that produce adrenocorticotrophic hormone (ACTH) are even rarer, with few reports in the literature about how to treat them [4]. Here, we report the case of 58-year-old male who developed Cushing’s syndrome (CS) secondary to ACTH production by a metastatic PNET and which was successfully controlled by a cytotoxic chemotherapy available in a setting of limited resources.

## Case report

**Figure 1.**
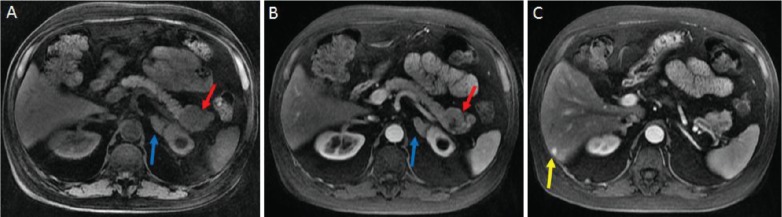
A: magnetic resonance imaging performed in the non-contrasting T1 sequence, showing an enlarged and irregular expansive lesion of the pancreas tail (red arrow) and thickening of the left adrenal (blue arrow). B: magnetic resonance imaging performed in the contrasting T1 sequence. C: nodular lesion in segment VI of the liver (yellow arrow).

In December 2013, a 58-year-old man with a history of type-2 diabetes mellitus (DM2), systemic arterial hypertension (SAH) and dyslipidemia was referred to our centre, the Hospital de Clínicas de Sao Paulo, a large academic and public centre located in Sao Paulo, Brazil, from the primary care service to investigate uncontrolled blood pressure and blood glucose. At initial clinical examination, he presented facial phletora, hirsutism, violaceous striae and centripetal obesity, and a CS clinical diagnosis was suspected. Initial examinations identified hypercortisolism and elevation of ACTH (Table 1). The ACTH increment in the corticotropin-releasing hormone (CRH) stimulation test was 14%, suggesting an ectopic ACTH-producing tumour. To confirm this hypothesis, the patient underwent an inferior petrosal sinus sampling with CRH stimulation. The central/peripheral ACTH gradient was less than 2 at 0, 3, 5 and 10 min after desmopressin injection, confirming the ectopic ACTH producing. Magnetic resonance image (MRI) of the abdomen showed an expansive lesion in the tail of the pancreas measuring 5.0 cm in the largest diameter, multiple small nodules in hepatic segment III and increased volume of both adrenals (Figure 1). In January 2014, he was referred to our institution, the Instituto do Cancer do Estado de Sao Paulo, where he underwent an R0 body-caudal pancreatectomy, splenectomy and removal of the segment III of the liver, which was compatible with PNET, infiltrating the liver tissue. The final diagnosis was of a well-differentiated PNET, with positive immunohistochemistry staining of synaptophysin and chromogranin A, three mitosis/10 high-power fields and Ki67 index of 6%. There was adequate clinical control of DM2 and SAH after resection. In July 2014, a Gallium-68-DOTATATE PET-CT was performed and did not show any measurable metastatic disease. However, in November 2014, he presented hyperglycaemia and high blood pressure again, associated with elevation of both serum cortisol and ACTH, which were resultant from hepatic recurrence with a new lesion of 1.2 cm in segment VI of the liver. Because the patient was quite sick to undergo new hepatic resection due to uncontrolled CS and also because of the short interval from last metastasectomy, new surgical resection was contraindicated. Octreotide LAR 20 mg IM once every 28 days was started but was unsuccessful in controlling symptoms. Subsequent hepatic embolisation did not improve his condition either. Due to uncontrolled CS in February 2015, bilateral adrenalectomy was performed and the CS finally resolved. The pathology report revealed a metastatic neuroendocrine tumour in the left adrenal, with immunohistochemistry staining positive for synaptophysin, chromogranin A positive and ACTH, negative staining for CD56, CDX2 and TTF1 negative and ki67 index of 30%.

**Figure 2.**
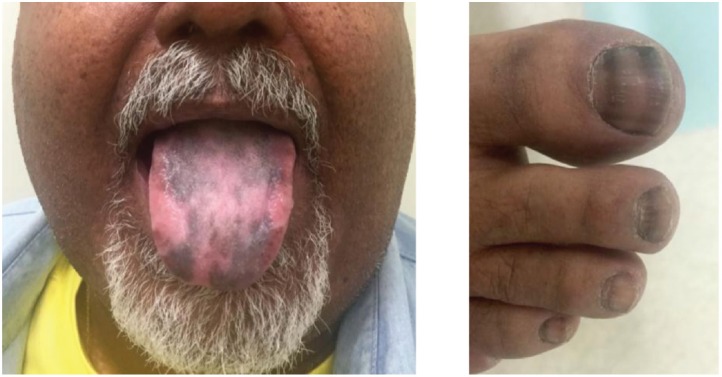
Hyperpigmented lesions of his tongue and nails likely caused by increased propiomelanocortin (POMC) prohormone production, which is cleaved into ACTH and MSH, inducing darkening of the skin and nails. The strips in his nails timely occurred when the tumour responded to CApOx

After symptom control, the patient was lost to follow up, returning six months with recurrent hyperglycaemia and skin hyperpigmentation; at that time, the elevation of plasma ACTH was identified. A new MRI of the abdomen showed a progression of hepatic metastases. Octreotide LAR was tried again, but there was disease progression after two doses. In October 2015, he received the combination of oxaliplatin 130 mg/m^2^ given on day one and capecitabine 1000 mg/m^2^ (CapOx) orally for 14 days, in a 21-days cycle. At this time, he presented diffuse exuberant hyperpigmented lesions of the skin, mainly in interphalangeal joints and tongue (Figure 2). After two cycles, there was significant improvement in cutaneous hyperpigmentation and the patient was restaged with new CT scans that showed stable disease. Despite good tolerance, the patient requested to stop chemotherapy and he went on chemoholiday. In March 2016, after three months without treatment, the hyperpigmented lesions of the skin worsened and imaging tests evidenced new progression of liver disease. Re-exposure to CapOx was indicated. He received three more cycles, when in in April/2016, CT scans demonstrated partial response in liver lesions. The patient chose to pause the chemotherapy once again. At the last image evaluation, in June 2017, the tumour has remained stabilised and three has been no further worsening of skin hyperpigmentation.

## Discussion

While the most frequent functional PNETs are insulinoma and gastrinoma, ectopic adrenocorticotropic hormone-producing tumours are very rare types of PNET [5, 6] and carry a poor prognosis. The survival analysis of all the cases reported in the English/Spanish literature found that only 35% of patients were alive at 5 years after diagnosis [7]. Maragliano et al have reported a series of 124 cases of ACTH-secreting PNETs, in which female patients predominated (66%), the mean age at diagnosis was 42.1 years, 45% arose from the tail of the pancreas and most tumours were well differentiated (94%) [7]. Up to 78.7% of ACTH-secreting PNETs have exhibited distant metastases when diagnosed, with the most common sites being liver and lungs followed by the adrenal glands, bone, kidney and omentum [8].

The ectopic ACTH syndrome is responsible for 10–15% of all causes of CS. The symptoms intensity depends on the cortisol serum levels and the tumour aggressiveness. The clinical manifestations are diverse, from incidental diagnosis in asymptomatic patients to classical CS features, with proximal muscle fatigue, striae, skin pigmentation, hypertension, abdominal pain and high susceptibility to infection. The most frequent tumours associated with ectopic production of ACTH are small-cell carcinoma of the lung, followed by pancreatic, bronchial carcinoid tumours, thymic carcinoid tumours, medullary thyroid carcinoma and pheochromocytoma [9–11].

Table 2 shows studies that evaluated Cushing’s syndrome in patients with PNETs. The treatment aim for ectopic CS associated with PNETs was the maximum biochemical control of hypercortisolaemia, often combined with antitumour therapy. Medical treatment for CS mostly relies on steroidogenesis inhibitors (ketoconazole, mitotane and metyrapone). The CS control with these drugs is usually partial and transitory. In addition, the steroidogenesis inhibitors are associated with a high rate of hepatotoxicity. In our experience, bilateral adrenalectomy is often the only effective intervention to control CS from ectopic-ACTH neuroendocrine tumours. Likewise experts tend to indicate bilateral adrenalectomy to treat severe cases of CS not properly controlled with medical treatment [4]. Surgery debulking can be recommended with the aim of tumour for symptom control; hepatic embolization and radiofrequency ablation are also reasonable therapeutic options, although there are no data about their efficacy in controlling CS from ACTH-producing PNETs. In patients with advanced, surgically non-resectable or progressive PNET, systemic treatment can be performed [12]. Treatment of metastatic disease may reduce neoplastic lesions and control the ACTH and cortisol production [13]. However, there are few data about the effects of systemic therapies to treat CS associated with NET.

Somatostatin analogue, octreotide and lanreotide can be used to control neoplastic growth and ACTH ectopic production in tumours with somatostatin receptors expression, which is found in 80% of PNET [14]. Acting in somatostatin receptor subtype-2, the somatostatin analogue-controlled ACTH levels in a case report [15]. But given that ACTH-producing PNET tend to be aggressive, somatostatin analogues, in our experience, do not provide long-term tumour or symptom control. Targeted therapy represented by everolimus and sunitibe have been approved for the treatment of advanced well-differentiated PNETs. The RADIANT-3 trial showed that everolimus provided a 11-month median progression-free survival benefit (PFS) versus 4.6 months in the placebo group [16]. Another phase-III study demonstrated that sunitinib led to a median PFS of 11.4 months versus 5.5 months in the placebo group [17]. In terms of symptom and biochemical control, small case series showed the benefit of everolimus to control hypoglycaemia caused by metastatic insulinomas [18] and to improve carcinoid symptoms in patients with midgut tumors, [19] but data on the symptom control of ACTH-producing PNETs are lacking. Other treatment modalities include the use of systemic chemotherapy and interferon. Various single and combinatory cytotoxic chemotherapeutic agents have been used in the treatment of PNET, but there is little data on the best form of treatment for ACTH-producing PNET.

In our institution, which is funded by the Brazilian Unified Health System, targeted therapies and peptide receptor radionuclide therapy for NET are not available. Instead, systemic chemotherapy is the only reimbursed option for metastatic PNET. Bajetta et al conducted a phase-II trial of patients with metastatic neuroendocrine tumours, demonstrating a response rate of 30 among the well-differentiated tumours [20]. CApOx is the standard chemotherapy regimen for PNET in our institution and our retrospective data showed that the use of CApOX in patients with metastatic PNET offered a median disease progression of 9.8 months, partial objective response of 29% and stable disease in 71% of cases [21] . However, there are very limited data on the efficacy of chemotherapy to treat hormonal-syndromes associated with functioning PNETs. As demonstrated in our case, the CapOx, an inexpensive treatment, was able to control not only the tumour but also its ACTH production, as evidenced by the improvement in skin and nail hyperpigmentation. This had a positive impact on the patient’s quality of life in terms of improved self-esteem resulting from the normalisation of his skin colour.

## Conclusion

Because of the rarity of ACTH-secreting PNETs, evidence-based treatment decisions are lacking, with the only source of scientific data being case series and reports. The experience of this case demonstrates that CApOX, an inexpensive regimen, may benefit patients with this tumour and represents a good option in settings of limited resources.

## Conflicts of Interest

The authors declare no conflict of interest.

## Figures and Tables

**Table 1.** Laboratory tests performed during treatment.

Test	03 December 2013	07 July 2014	17 November 2014	14 July 2015
Seric cortisol (6.7 a 22.6 µg/dL)	48	13.8	25.8	10.8
Urinary cortisol (50 a 310 µg/24h)	6519		3360	
ACTH ( < 46 pg/mL)	190	17	195.6	1992.0
Seric glucose (70 a 100 mg/dL)	289	185	206	263.0
Glycated hemoglobina (4.1 a 6.0 %)	10.6	8.8	7.9	10.1

The first column shows the elevation of serum and urinary cortisol levels and serum ACTH levels at diagnosis, as well as values confirming a decompensated type-2 diabetes mellitus. The second column shows improvement in hormonal and glycaemic levels after debulking surgery of the pancreas and liver. The third and fourth column shows the values when we evidenced disease progression to the liver in two different moments of his treatment.

**Table 2.** Cases series dealing with ectopic ACTH secretion with information on patient treatment and study outcomes.

Author/Year of publication	Study design	*N*	Medical treatment	Particularities and outcomes
Ilias I *et al* 2005	Retrospective	90	Sixty-two patients received steroidogenesis inhibitors: ketoconazole, metyrapone, aminoglutethimide, etomidate, among others and were discontinued or changed because of side effects or inadequate inhibition. To control hypercortisolism, 33 patients underwent bilateral or completion adrenalectomy.	Of the 90 patients only one had PNET and 13 had other types of NETs. Surgical resection of the corticotropin-secreting tumour was achieved in 59 of 90 patients (65%) and 42 were cured.
Isidori AM *et al*. 2005	Retrospective	40	Twenty-eight patients received steroidogenesis inhibitors for periods of 4 wk to 96 months (median, 9 months). One patient required iv treatment with etomidate to control the hypercortisolemia. Bilateral adrenalectomy was performed in 12 patients. 12 patients underwent an attempt at curative resection.	The median follow-up was 5 years. 3/40 patients had PNETs. Bronchial carcinoid tumours were the most common cause of EAS*, followed by other neuroendocrine tumours. Tumour histology and, in the subgroup of NETs, the presence of non-lymph node metastases, were the most important prognostic factors predicting overall survival (*p* < 0.05).
Maragliano R *et al*. 2015	Cases series	11	Only clinical and histologic descriptions are provided	Most are well-differentiated NETs but woth histological features of aggressiveness, with ki67 index ranging from 3% to 20%.
Reincke M *et al*. 2015	Retrospective	248	Bilateral adrenalectomy	Thirty patients had ectopic Cushing’s syndrome. Mortality was higher in studies that involved patients with ectopic Cushing’s syndrome than it is in patients with Cushing’s disease (surgical mortality 5.7 vs. 2.4%, respectively; total mortality 46.2% vs. 10.2%, respectively).
Kamp K *et al*. 2016	Retrospective	918	Twenty-three patients with EAS received medical treatment with mifepristone, ketoconazole, etomidate and somatostatin analogues. There was poor control of hypercortisolemia with these drugs and 15 of these patients underwent bilateral adrenalectomy. Curative resection or surgical debulking of was performed in 16 patients with EAS. Seven patients were treated with peptide receptor radiotherapy, six patients (3 PNETs) were treated with a median dose of Lu^177^-octreotate of 29.0 GBq. Other adjunctive treatments were cytotoxic chemotherapy (10.3%) and everolimus (6.9%).	The prevalence of EAS in a large cohort of patients with sporadic thoracic and GEP-NETs was 3.2%. 305 PNETs were identified and 10 patients had EAS*. Median OS of non-EAS thoracic and GEP-NET** patients with EAS was 61.2 months and 41.4 months (*p* = 0.151), but five-year survival was shorter in patients with EAS compared with non-EAS patients (*p* = 0.013). Lu^177^-octreotate was used as an adjuvant treatment to surgery, which resulted in two complete responses
Davi MV *et al*. 2017	Retrospective multicentre	110	Ketoconazole, mitotane, metyrapone, mifepristone, cabergoline, octreotide, lanreotide, 54.5% had surgery, 28.2% patients underwent adrenalectomy, everolimus, sunitinib (8.2%) and/or chemotherapy (24.5%)	The ectopic ACTH tumours were bronchial carcinoids (40.9%), occult tumours (22.7%) and PNET (15.5%). At diagnosis, 76% of PNET patients presented metastases. Overall survival rate at five years, without adjusting for stages, was 60% for PNET and 86% for bronchial carcinoids (*p* = 0.033). Negative predictive factors for survival in univariate analyses (no multivarable analyses reported) were severity of hypercortisolism, the presence of either hypokalaemia, diabetes mellitus and/or distant metastases. Partial or complete hormonal control with peptide redionuclide receptor therapy was achieved in 76.9% of patient

* EAS: ectopic ACTH syndrome.

** GEP-NET: gastroenteropancreatic neuroendocrine tumours.
